# Polymorphisms in the Inflammatory Pathway Genes *TLR2*, *TLR4*, *TLR9*, *LY96*, *NFKBIA*, *NFKB1*, *TNFA*, *TNFRSF1A*, *IL6R*, *IL10*, *IL23R*, *PTPN22*, and *PPARG* Are Associated with Susceptibility of Inflammatory Bowel Disease in a Danish Cohort

**DOI:** 10.1371/journal.pone.0098815

**Published:** 2014-06-27

**Authors:** Steffen Bank, Paal Skytt Andersen, Johan Burisch, Natalia Pedersen, Stine Roug, Julie Galsgaard, Stine Ydegaard Turino, Jacob Broder Brodersen, Shaista Rashid, Britt Kaiser Rasmussen, Sara Avlund, Thomas Bastholm Olesen, Hans Jürgen Hoffmann, Marianne Kragh Thomsen, Vibeke Østergaard Thomsen, Morten Frydenberg, Bjørn Andersen Nexø, Jacob Sode, Ulla Vogel, Vibeke Andersen

**Affiliations:** 1 Medical Department, Viborg Regional Hospital, Viborg, Denmark; 2 Biomedicine, University of Aarhus, Aarhus, Denmark; 3 Microbiology and Infection Control, Statens Serum Institut, Copenhagen, Denmark; 4 Department of Gastroenterology, Herlev Hospital, Herlev, Denmark; 5 Department of Gastroenterology, Hvidovre Hospital, Hvidovre, Denmark; 6 Medical Department, Køge Hospital, Køge, Denmark; 7 Medical Department, Hillerød Hospital, Hillerød, Denmark; 8 Medical Department, Sydvestjysk Hospital, Esbjerg, Denmark; 9 Medical Department, Bispebjerg Hospital, Bispebjerg, Denmark; 10 Medical Department, Nykøbing Falster Hospital, Nykøbing Falster, Denmark; 11 Medical Department V, Aarhus University Hospital, Aarhus, Denmark; 12 Medical Department, Slagelse Hospital, Slagelse, Denmark; 13 Department of Respiratory Diseases B, Institute for Clinical Medicine, Aarhus University Hospital, Aarhus, Denmark; 14 Department of Clinical Microbiology, Aarhus University Hospital, Aarhus, Denmark; 15 International Reference Laboratory of Mycobacteriology, Statens Serum Institut, Copenhagen, Denmark; 16 Section of Biostatistics, Department of Public health, Aarhus University, Aarhus, Denmark; 17 Institute of Regional Health Research, University of Southern Denmark, Odense, Denmark; 18 Clinical Biochemistry, Immunology & Genetics, Statens Serum Institut, Copenhagen, Denmark; 19 Department of Rheumatology, Frederiksberg Hospital, Frederiksberg, Denmark; 20 National Research Centre for the Working Environment, Copenhagen, Denmark; 21 Organ Centre, Hospital of Southern Jutland Aabenraa, Aabenraa, Denmark; 22 OPEN Odense Patient data Explorative Network, Odense University Hospital, Odense, Denmark; Charité, Campus Benjamin Franklin, Germany

## Abstract

**Background:**

The inflammatory bowel diseases (IBD), Crohn's disease (CD) and ulcerative colitis (UC), result from the combined effects of susceptibility genes and environmental factors. Polymorphisms in genes regulating inflammation may explain part of the genetic heritage.

**Methods:**

Using a candidate gene approach, 39 mainly functional single nucleotide polymorphisms (SNPs) in 26 genes regulating inflammation were assessed in a clinical homogeneous group of severely diseased patients consisting of 624 patients with CD, 411 patients with UC and 795 controls. The results were analysed using logistic regression.

**Results:**

Sixteen polymorphisms in 13 genes involved in regulation of inflammation were associated with risk of CD and/or UC (p≤0.05). The polymorphisms *TLR2* (rs1816702), *NFKB1* (rs28362491), *TNFRSF1A* (rs4149570), *IL6R* (rs4537545), *IL23R* (rs11209026) and *PTPN22* (rs2476601) were associated with risk of CD and the polymorphisms *TLR2* (rs1816702), *TLR4* (rs1554973 and rs12377632), *TLR9* (rs352139), *LY96* (rs11465996), *NFKBIA* (rs696), *TNFA* (rs1800629), *TNFRSF1A* (rs4149570), *IL10* (rs3024505), *IL23R* (rs11209026), *PTPN22* (rs2476601) and *PPARG* (rs1801282) were associated with risk of UC. When including all patients (IBD) the polymorphisms *TLR2* (rs4696480 and rs1816702), *TLR4* (rs1554973 and rs12377632), *TLR9* (rs187084), *TNFRSF1A* (rs4149570), *IL6R* (rs4537545), *IL10* (rs3024505), *IL23R* (rs11209026) and *PTPN22* (rs2476601) were associated with risk. After Bonferroni correction for multiple testing, both the homozygous and the heterozygous variant genotypes of *IL23R* G>A(rs11209026) (OR_CD,adj_: 0.38, 95% CI: 0.21–0.67, p = 0.03; OR_IBD,adj_ 0.43, 95% CI: 0.28–0.67, p = 0.007) and *PTPN22* 1858 G>A(rs2476601) (OR_CD,unadj_ 0.54, 95% CI: 0.41–0.72, p = 7*10^−4^; OR_IBD,unadj_: 0.61, 95% CI: 0.48–0.77, p = 0.001) were associated with reduced risk of CD.

**Conclusion:**

The biological effects of the studied polymorphisms suggest that genetically determined high inflammatory response was associated with increased risk of CD. The many SNPs found in *TLR*s suggest that the host microbial composition or environmental factors in the gut are involved in risk of IBD in genetically susceptible individuals.

## Introduction

Chronic inflammatory bowel diseases (IBDs), Crohn's disease (CD) and ulcerative colitis (UC), are complex diseases that result from the interaction of numerous genetic and environmental factors [1].

Genetic association studies have identified innate immunity as a critical component in the development of IBD. Until now, more than 163 IBD susceptibility polymorphisms have been confirmed, most of which are associated with both CD and UC, by candidate and genome wide association studies (GWAS) [2]–[11]. However, these polymorphisms have been estimated to only account for 20% of the genetic heritage involved in IBD [12].

Functional polymorphisms in genes in the inflammatory pathways may explain some of the genetic heritage involved in IBD. The transcription factor NFκB is a central regulator of inflammation. NFκB can be activated by Toll like receptors (TLRs). TLRs recognize pathogen-associated molecular patterns (PAMPs) that are broadly shared by pathogens but distinguishable from host molecules such as bacterial or viral DNA, flagellin or lipopolysaccharide (LPS). The TLRs initiates a kinase cascade that ultimately activates the IKK-complex, which phosphorylates and degrades the NFκB inhibitor IκBα. NFκB is shuttled from the cytosol to the nucleus where it initiates expression of pro- and anti-inflammatory cytokines including TNF-α, IL-6 and IL-10 [13].

To identify susceptibility loci we assessed 39 mainly functional polymorphisms in genes involved in inflammation, particular in the NFκB pathway, in a homogeneous Danish cohort of 624 patients with severe CD, 411 patients with severe UC and 795 healthy controls. The candidate gene approach using functional polymorphisms allows interpretation of the underlying biological mechanisms based on increased or decreased gene expression or protein activity.

Functional polymorphisms in genes in the inflammatory pathways involved in regulation of the NFκB pathway (*TLR2*, *TLR4*, *TLR5*, *TLR9*, *LY96*, *CD14*, *MAP3K14*, *SUMO4*, *NFKBIA* and *NFKB1*), TNF-α signaling (*TNFA*, *TNFRSF1A* and *TNFAIP3*), cytokines regulated by NFκB (*IL1B*, *IL1RN*, *IL6*, *IL10*, *IL17A* and *IFNG*) and other genes involved in regulation of inflammation (*IL4R*, *IL6R*, *IL23R*, *TGFB1*, *PTPN22*, *PPARG* and *NLRP3*) were studied, as genetically determined variation in the inflammatory pathways may be associated with severe disease among patients with CD and UC.

## Materials and Methods

### Cohort

A prior anti-TNF naïve Danish cohort of patients with IBD was established. In short, blood samples retrieved as part of the routine screening for latent *Mycobacterium tuberculosis* at Statens Serum Institut (SSI, Copenhagen, Denmark) and the Department of Respiratory Diseases B or the Department Clinical Microbiology, Aarhus University Hospital (Aarhus, Denmark) were collected from 01.09.2009 to 30.03.2011 (9217 patients). Patients with intestinal diseases (ICD-10 code K50–K63) were identified by linking the unique personal identification number of Danish citizens (CPR-number) from each blood sample with the National Patient Registry (2659 patients). Patient records from 18 medical departments were examined (1378 patients) and identified 1035 ethnic Danish patients with IBD where blood and clinical data were available. The patients either received or were considered candidates to anti-tumor necrosis factor-α (TNF-α) therapy (infliximab or adalimumab). The control group consisted of 795 healthy blood donors recruited from Viborg, Denmark [4].

### Selection of polymorphisms

A candidate gene approach was used with focus on polymorphisms in the TNF-α and NFκB pathways. In addition, polymorphisms in genes which have been shown to be associated with CD and/or UC, polymorphisms in inflammatory cytokines and polymorphisms in *TLR2* and *TLR4* were included [14].

Functional polymorphisms in relevant genes were found by searching pubmed with “polymorphism AND gene-name AND (reporter gene OR luciferase OR ELISA OR enzyme-linked immunosorbent assay OR RT-PCR OR reverse transcriptase PCR OR EMSA OR electrophoretic mobility shift assay OR flow cytometry)”.

### Genotyping

For patients with IBD the DNA was extracted from cryopreserved blood clots by using the Maxwell 16 Blood purification kit (Promega) according to the manufacturers' instructions with a median yield of 4.90 µg (range 0.8–25 µg) pr 300 µl total blood [15]. For the healthy controls, DNA was extracted from EDTA-stabilized peripheral blood by either PureGene (Qiagen, Hilden, Germany) or Wizard Genomic (Promega, Madison, Wisconsin, USA) DNA purification kit according to the manufacturers' instructions [4]. Competitive Allele-Specific Polymerase chain reaction (KASP), an end-point PCR technology, was used by LGC Genomics for genotyping (LGC Genomics , Hoddesdon, United Kingdom) (http://www.lgcgenomics.com/). The SNPs studied were *TLR2* (rs4696480, rs1816702, rs11938228, rs3804099), *TLR4* (rs12377632, rs5030728, rs1554973), *TLR5* (rs5744168), *TLR9* (rs187084, rs352139), *LY96* (MD-2) (rs11465996), *CD14* (rs2569190), *MAP3K14* (NIK) (rs7222094), *SUMO4* (rs237025), *NFKBIA* (IκBα) (rs696, rs17103265), *NFKB1* (NFκB1) (rs28362491), *TNFA* (TNF-α) (rs1800629, rs1800630, rs1799724, rs361525), *TNFRSF1A* (TNFR1) (rs4149570), *TNFAIP3* (A20) (rs6927172), *IL1B* (IL-1β) (rs1143623, rs4848306, rs1143627), *IL-1RN* (IL-1RA) (rs4251961), *IL4R* (rs1805010), *IL6* (rs10499563), *IL6R* (rs4537545), *IL10* (rs1800872, rs3024505), *IL17A* (rs2275913), *IL23R* (rs11209026), *IFNG* (IFN-γ) (rs2430561), *TGFB1* (TGF-β1) (rs1800469), *PTPN22* (rs2476601), *PPARG* (PPAR-γ) (rs1801282) and *NLRP3* (rs4612666).

Genotyping of *TNFA* (TNF-α) −857 C>T (rs1799724) and −863 C>A (rs1800630) failed due to their close proximity to each other. All genotyping of −857 C>T (rs1799724) either failed or were erroneously genotyped as homozygous wild type when the patients were carriers of the AA genotype of −863 C>A (rs1800630) due to genotyping bias.

The 39 genotypes were replicated in 94 randomly selected samples and yielded >99% identical genotypes.

### Statistical analysis

Logistic regression was used to compare genotype distributions among patients with CD, UC and IBD versus healthy controls (Table S1 and S2). Crude odds ratio and odds ratio adjusted for age, gender and smoking status were assessed. A chi-square test was used to test for deviation from Hardy-Weinberg equilibrium in the healthy controls and for haplotype analysis (Table S3, S4, S5).

Statistical analyses were performed using STATA version 11 (STATA Corp., Texas, USA).

### Ethics statement

The study was conducted in accordance with the Declaration of Helsinki and was approved by the Regional Ethics Committees of Central (M20100153) and Southern (S-20120113) Denmark and the Danish Data Protection Agency of Central (RM: J. 2010-41-4719) and Southern (RSD: 2008-58-035) Denmark. The Ethics Committees gave suspension for obtaining written informed consent.

## Results

### Study population

Characteristics of the Danish patients with CD, UC and healthy controls are shown in Table 1.

**Table 1:** Description of the study participants.

	Crohns Disease (CD)	Ulcerative Colitis (UC)	Controls
	(n = 624)	(n = 411)	(n = 795)
**Gender: n (%)**			
Male	272 (44)	201 (49)	411 (52)
Female	352 (56)	210 (51)	384 (48)
**Age:**			
Median (5%–95%)	37 (20–67)	42 (20–72)	43 (23–60)
**Age at diagnosis:**			
Median (5%–95%)	25 (14–59)	33 (15–67)	-
**Smoking habits: n (%)**			
Smokers	178 (29)	30 (7)	207 (26)
Former smokers	64 (10)	86 (21)	392 (49)
Never smokers	156 (25)	102 (25)	189 (24)
Data not available	226 (36)	193 (47)	7 (1)
**Location UC: n (%)**			
Proctitis (E1)	-	53 (13)	-
Left side (E2)	-	183 (45)	-
Extensive (E3)	-	134 (33)	-
Data not available	-	41 (10)	-
**Location CD: n (%)**			
Colonic (L2)	208 (33)	-	-
Ileal (L1)	172 (28)	-	-
Ileocolonic (L3)	210 (34)	-	-
Data not available	34 (5)	-	-

The genotype distributions among the healthy controls deviated from Hardy-Weinberg equilibrium for *TLR2* (−16934 A>T (rs4696480)) (p = 0.02), *TLR4* (rs1554973 T>C) (p = 0.03), *TLR9* (1174 G>A (rs352139)) (p = 0.02), and *TGFB1* (−509 C>T (rs1800469)) (p = 0.02). None of the deviations remained statistically significant after correction for multiple testing.

### Polymorphisms associated with risk of CD

The homozygous variant genotype of *TLR2* C>T (rs1816702) (OR_adj_: 2.80, 95% CI: 1.03–7.62, p = 0.04), *TNFRSF1A* −609 G>T (rs4149570) (OR_adj_: 1.84, 95% CI: 1.19–2.84, p = 0.01) and *IL6R* C>T (rs4537545) (OR_adj_: 1.73, 95% CI: 1.12–2.66, p = 0.01) were associated with increased risk of CD. Both the homozygous and the heterozygous variant genotypes of *NFKB1* −94ins/del (rs28362491) (OR_unadj_: 0.80, 95% CI: 0.65–1.00, p = 0.05), *IL23R* G>A (rs11209026) (OR_adj_: 0.38, 95% CI: 0.21–0.67, p = 9*10^−4^) and *PTPN22* 1858 G>A (rs2476601) (OR_adj_: 0.57, 95% CI: 0.39–0.83, p = 4*10^−3^) were associated with reduced risk of CD (Table S1 and S2).

After Bonferroni correction for multiple testing both the homozygous and the heterozygous variant genotypes of *IL23R* G>A (rs11209026) (OR_adj_: 0.38, 95% CI: 0.21–0.67, p = 0.03) and *PTPN22* 1858 G>A (rs2476601) (OR_unadj_: 0.54, 95% CI: 0.41–0.72, p = 7*10^−4^) were associated with reduced risk of CD.

The variant allele of the polymorphisms have been shown to increase TLR2 levels (*TLR2* C>T (rs1816702)), increase *TNFRSF1A* expression (*TNFRSF1A* −609 G>T (rs4149570)), increase IL-6r and IL-6 levels (*IL6R* C>T (rs4537545)), decrease NF-κB p50 subunit expression (*NFKB1* −94ins/del (rs28362491)), decrease IL-17 serum levels (*IL23R* G>A (rs11209026)) and decrease TNF-α serum levels (*PTPN22* 1858 G>A (rs2476601)), respectively (Table 2).

**Table 2:** The biologic effect of the studied single nucleotide polymorphism (SNP) and odds ratios (OR) for polymorphisms which have been shown to be associated with risk of Crohn's disease (CD), ulcerative colitis (UC) or inflammatory bowel disease (IBD) in previous studies and in this study.

Gene (SNP)	rs-number	Effect of the SNP	Previously found associations. Disease, genotype, OR (95% CI), p-value	Associations found in this study. Disease, genotype, OR (95% CI), p-value
*TLR2* (activates inflammation through the canonical NFκB pathway)				
−16934 A>T	rs4696480	Unknown [14]	ND	IBD: TT, 1.33 (1.01–1.74), p = 0.04^A^
C>A	rs11938228	Unknown [14]	ND	No association
C>T	rs1816702	rs1816702T increase receptor level^C^ [31]	ND	CD: TT, 2.36 (1.08–5.16), p = 0.03^A^^,^ B; UC: CT or TT, 1.46 (1.10–1.93), p = 0.009^A^^,^ B; IBD: CT or TT, 1.32 (1.05–1.65), p = 0.02^A^^,^ B
597 T>C	rs3804099	597C decrease TNF-α, IL-1β & IL-6 level^E^ [32]	ND	No association
*TLR4* (activates inflammation through the canonical or non-canonical NFκB pathway)				
G>A	rs5030728	Unknown [14]	ND	No association
T>C	rs1554973	Unknown [14]	ND	UC: TC or CC, 0.67 (0.48–0.94), p = 0.02^B^; IBD: CC, 0.67 (0.46–0.98), p = 0.04^A^
T>C	rs12377632	Unknown [14]	ND	UC: TC or CC, 1.42 (1.01–2.00), p = 0.04^B^; IBD: TC or CC, 1.28 (1.01–1.64), p = 0.05^B^
*TLR5* (activates inflammation through the canonical NFκB pathway)				
1174 C>T	rs5744168	1174T (392^STOP^), decrease TNF-α, IL-1β & IL-6 level^G^ [32] and inhibit TLR5 function^D^^,^ E [33]	CD: CT, 0.14 (0.03–0.57), p = 0.002 (Jewish) [28]; CD: No association (Non-Jewish) [28], [29]	No association
*TLR9* (activates inflammation through the canonical NFκB pathway)				
−1486 T>C	rs187084	−1486C&1174G decrease expression^D^ [34]	ND	IBD: TC or CC, 1.29 (1.00–1.66), p = 0.05^B^
1174 G>A	rs352139	−1486C&1174G decrease expression^D^ [34]	ND	UC: AA, 0.67 (0.47–0.95), p = 0.03^A^
*LY96* (MD-2 binds to and is involved in the TLR2 or the TLR4 complexes)				
−1625 C>G	rs11465996	−1625G increase MD-2 & TNF-α level^D^^,^ E [35]	ND	UC: CG, 0.74 (0.57–0.96), p = 0.02^A^
*CD14* (binds LPS and transport it to TLR4)				
−159 G>A	rs2569190	−159AA increase CD14 level^E^ [36], [37]	IBD: GA or AA, 2.95(1.77–4.90),p = 2*10^−5^ [22] (Korean)	No association
*MAP3K14* (NIK is a central kinase in the non-canonical NFκB pathway)				
T>C	rs7222094	rs7222094CC decrease NIK activity^E^ [38]	ND	No association
*SUMO4* (SUMO4 conjugates to IκBα and negatively regulates NFκB transcriptional activity)				
163 T>C	rs237025	163C increase NFκB1 expression^D^ [39]	ND	No association
*NFKBIA* (IkBα is an inhibitor of NFκB1)				
2758 G>A	rs696	2758A increase expression^D^ [40]	UC:Extensive colitis (Hungarian) [23]; CD: Inconclusive [24], [25]	UC: GA or AA, 1.28 (1.00–1.65), p = 0.05^A^^,^ B; CD: No association
T>del	rs17103265	rs17103265del decrease expression^D^ [41]	ND	No association
*NFKB1* (NFκB1 (p50/65) is a transcription factor. The NF-κB p50 subunit can act both pro-inflammatory as part of the p50/p65 complex or anti-inflammatory as p50 homodimer [42])				
−94 ins/del	rs28362491	−94del decrease p50 subunit expression^D^^,^ G [43]	Inconclusive [26]	CD: Ins/- or -/- , 0.80 (0.65–1.00), p = 0.05^A^
*TNFA* (TNF-α is a pro-inflammatory cytokine activated by NFκB1)				
−863 C>A	rs1800630	−863A increase expression^D^^,^ G [44]	IBD: AA, 4.82 (2.60–8.96), p = 1*10^−4^ (Indian) [45]	Failed to genotype
−857 C>T	rs1799724	−857T increase TNF-α level^D^^,^ E ^,^ F [46]	Inconclusive [27]	Failed to genotype
−308 G>A	rs1800629	−308A increase expression^C^^,^ D [47]	Inconclusive [27]	UC: GA or AA, 0.75 (0.58–0.98), p = 0.04^A^
−238 G>A	rs361525	−238A decrease expression^D^^,^ E [48]	Inconclusive [27]	No association
*TNFRSF1A* (TNF receptor 1 (TNFR1) binds TNF-α and initiates a kinase cascade)				
−609 G>T	rs4149570	−609T increase expression^F^ [49]	ND	CD: TT, 1.41 (1.02–1.94), p = 0.04^A^^,^ B; UC: TT, 1.49 (1.04–2.13), p = 0.03^A^^,^ B; IBD: GT or TT, 1.23 (1.02–1.50), p = 0.03^A^^,^ B
*TNFAIP3* (TNF-α rapidly induced expression of *TNFAIP3*/A20 which inhibit NFκB activation and TNF-α mediated apoptosis)				
C>G	rs6927172	rs6927172G increase expression^D^^,^ G [50]	ND	No association
*IL1B* (pro-inflammatory cytokine activated by NFκB1)				
−3737 G>A	rs4848306	−3737A decrease transcription^F^ [51], [52]	ND	No association
−1464 G>C	rs1143623	rs1143623C decrease IL-1b level^E^ [52], [53]	ND	No association
−31 T>C	rs1143627	−31C decrease expression^D^^,^ E ^,^ G [52]–[54]	No association (Danish) [3]	No association
*IL1RN* (IL-1RA binds to the IL-1 receptor and inhibit IL-1β signaling)				
T>C	rs4251961	rs4251961C decrease IL-1RA level^E^ [55], [56]	ND	No association
*IL4R* (IL-4 receptor, IL-4 significantly inhibit IL-17 production)				
A>G (I50V)	rs1805010	rs1805010G increase IL-17 level^C^^,^ E [57]	ND	No association
*IL6* (pro- and anti-inflammatory cytokine activated by NFκB1)				
−6331 T>C	rs10499563	−6331C decrease expression^D^^,^ G [58]	ND	No association
*IL6R* (binds IL-6 and initiates a kinase cascade)				
C>T	rs4537545	rs4537545TT increase IL-6r and IL-6 level but not TNF-α, IL-1RA and CRP level^E^ [59]	ND	CD: TT, 1.73 (1.12–2.66), p = 0.01^B^; IBD: TT, 1.46 (1.02–2.08), p = 0.04^B^
*IL10* (activated by NFκB1, capable of inhibiting synthesis of pro-inflammatory cytokines such as IFN-γ and TNF-α)				
−592 C>A	rs1800872	−592A increase expression^D^ [60]	No association (Danish) [3]	No association
C>T	rs3024505	Unknown [3]	CD: T-allele, 1.12 (1.07–1.17),p = 2*10^−14^ [3], [16], [61]; UC: T-allele, 1.25 (1.19–1.32), p = 6*10^−17^ [3], [17]	CD: No association; UC: CT or TT, 1.42 (1.10–1.82), p = 0.007^A^; IBD: CT or TT, 1.25 (1.02–1.52), p = 0.03^A^
*IL17A* (activated by NFκB1, pro-inflammatory cytokine, potent mediator in delayed-type reactions, induces the production of IL-1β, IL-6 and TNF-α)				
197G>A	rs2275913	197A increase expression^D^^,^ F ^,^ G [62]	ND	No association
*IL23R* (IL-23 receptor, IL-23 induce the production of IL-17 and IFN-γ)				
G>A (R381Q)	rs11209026	rs11209026GG increase IL-17 serum level^E^ [63]	CD: G-allele, 2.66 (2.36–3.00), p = 1*10^−64^ [16]; UC: G-allele, 1.74 (1.57–1.92) , p = 5*10^−28^ [17]	CD: GA or AA, 0.39 (0.26–0.59), p = 1*10^−5A,^ B; UC: GA or AA, 0.59 (0.39–0.90), p = 0.01^A^^,^ B; IBD: GA or AA, 0.47 (0.34–0.66), p = 9*10^−6A,^ B
*IFNG* (IFN-γ is a pro- and anti-inflammatory cytokine activated by NFκB1)				
874 T>A	rs2430561	874A decrease IFN-γ level^E^ [64]	ND	No association
*TGFB1* (TGF-β1 is a cytokine which can inhibit the secretion and activity of many other cytokines including IFN-γ and TNF-α)				
−509 C>T	rs1800469	−509T increase expression^D^^,^ G [65]	ND	No association
*PTPN22* (involved in several signaling pathways associated with the immune response)				
1858 G>A	rs2476601	1858A decrease TNF-α in serum level^E^^,^ F [66]	CD: G-allele, 1.26 (1.17–1.37), p = 5*10^−9^ [16]	CD: GA or AA, 0.54 (0.41–0.72), p = 2*10^−5A,^ B; UC:GA or AA, 0.71 (0.52–0.96), p = 0.03^A^; IBD: GA or AA, 0.61 (0.48–0.77), p = 4*10^−5A,^ B
*PPARG* (PPARγ is a transcription factor)				
C>G (Pro12Ala)	rs1801282	rs1801282G decrease PPARγ mRNA level, but upregulations MyD88 TLR4, TLR5, TLR9, P65 and TNF-α mRNA levels^E^^,^ F [67]	CD:GG, 0.33 (0.12–0.94), p = 0.03 (Hungarian) [68]; CD: No association (Danish) [18]; UC: GG, 2.30 (1.04–5.08), p = 0.04 (Danish) [18]	CD: No association; UC: GG, 2.12 (1.01–4.45), p = 0.05^A^
*NLRP3* (NALP3 is involved in the inflammasome)				
C>T	rs4612666	rs4612666T decrease expression^D^ [69]	ND	No association

A Crude (unadjusted).

B Adjusted for age, gender and smoking status.

C Function examined by flow cytometry.

D Function examined by luciferase reporter assay.

E Function examined by enzyme-linked immunosorbent assay (ELISA).

F Function examined by reverse transcriptase PCR (RT-PCR).

G Function examined by electrophoretic mobility shift assay (EMSA).

ND: not determined.

Thus, polymorphisms associated with higher TLR2 levels (*TLR2* C>T (rs1816702)), increased *TNFRSF1A* expression (*TNFRSF1A* −609 G>T (rs4149570)) and higher IL-6r and IL-6 levels (*IL6R* C>T (rs4537545)) were associated with increased risk of CD. In addition, decreased IL-17 serum levels (*IL23R* G>A (rs11209026)) and decreased TNF-α serum levels (*PTPN22* 1858 G>A (rs2476601)) were associated with reduced risk of CD. The NF-κB p50 subunit can act both pro-inflammatory as part of the p50/p65 complex or anti-inflammatory as p50 homodimer (Table 2). A lowered NF-κB p50 subunit expression (*NFKB1* −94ins/del (rs28362491)) could be a differential lowering of the anti-inflammatory response which was associated with reduced risk of CD.

### Polymorphisms associated with risk of UC

The homozygous variant genotype of *TNFRSF1A* −609 G>T (rs4149570) (OR_adj_: 1.85, 95% CI: 1.16–2.97, p = 0.01) and *PPARG* C>G (rs1801282) (OR_unadj_: 2.12, 95% CI: 1.01–4.45, p = 0.05) and both the homozygous and the heterozygous variant genotypes of *TLR2* C>T (rs1816702) (OR_adj_: 1.63, 95% CI: 1.13–2.36, p = 0.01), *TLR4* T>C (rs12377632) (OR_adj_: 1.42, 95% CI: 1.01–2.00, p = 0.04), *NFKBIA* 2758 A>G (rs696) (OR_adj_: 1.45, 95% CI: 1.03–2.05, p = 0.03) and *IL10* C>T (rs3024505) (OR_unadj_: 1.42, 95% CI: 1.10–1.82, p = 0.01) were associated with increased risk of UC.

The homozygous variant genotype of *TLR9* 1174 G>A (rs352139) (OR_unadj_: 0.67, 95% CI: 0.47–0.95, p = 0.03) and the heterozygous genotype of *LY96* −1625 C>G (rs11465996) (OR_unadj_: 0.74, 95% CI: 0.57–0.96, p = 0.02) were associated with reduced risk of UC. In addition, both the homozygous and the heterozygous variant genotypes of *TLR4* T>C (rs1554973) (OR_adj_: 0.67, 95% CI: 0.48–0.94, p = 0.02), *TNFA* −308 G>A (rs1800629) (OR_unadj_: 0.75, 95% CI: 0.58–0.98, p = 0.04), *IL23R* G>A (rs11209026) (OR_adj_: 0.52, 95% CI: 0.29–0.94, p = 0.03) and *PTPN22* 1858 G>A (rs2476601) (OR_unadj_: 0.71, 95% CI: 0.52–0.96, p = 0.03) were associated with reduced risk of UC (Table S1 and S2). No associations were found after Bonferroni correction for multiple testing.

The variant allele of the polymorphisms have been shown to increase *TNFRSF1A* expression (*TNFRSF1A* −609 G>T (rs4149570)), increase TLRs and TNF-α mRNA levels (*PPARG* C>G (rs1801282)), increase TLR2 levels (*TLR2* C>T (rs1816702)), increase *NFKBIA* expression (*NFKBIA* 2758 A>G (rs696)), increase MD-2 (*LY96*) and TNF-α levels (*LY96* −1625 C>G (rs11465996)), increases *TNFA* expression (*TNFA* −308 G>A (rs1800629)), decrease IL-17 serum levels (*IL23R* G>A (rs11209026)) and decrease TNF-α serum levels (*PTPN22* 1858 G>A (rs2476601)), respectively (Table 2). The biological function of the polymorphisms *TLR4* T>C (rs12377632), *TLR4* T>C (rs1554973), *TLR9* 1174 G>A (rs352139) and *IL10* C>T (rs3024505) are unknown.

Thus, polymorphisms associated with increased *TNFRSF1A* expression (*TNFRSF1A* −609 G>T (rs4149570)), increased TLRs and TNF-α mRNA levels (*PPARG* C>G (rs1801282)) and increased TLR2 levels (*TLR2* C>T (rs1816702)) were associated with increased risk of UC. Furthermore, polymorphisms associated with decreased IL-17 serum levels (*IL23R* G>A (rs11209026)) and decreased TNF-α serum levels (*PTPN22* 1858 G>A (rs2476601)) were associated with reduced risk of UC. In contrary, lower activity of the NFκB pathway through increased *NFKBIA* (IκBα) expression (*NFKBIA* 2758 A>G (rs696)), an inhibitor of the NFκB pathway, was associated with increased risk of UC. In addition, polymorphisms associated with increased MD-2 (*LY96* −1625 C>G (rs11465996)) and TNF-α levels (*TNFA* −308 G>A (rs1800629)) were associated with reduced risk of UC.

### Polymorphisms associated with risk of IBD

The studied polymorphisms all showed the same direction of effect for both diseases except for the polymorphism in *PPARG* (rs1801282) (Table S1 and S2).

When including all patients (IBD), the homozygous variant genotype of *TLR2* A>T (rs4696480) (OR_unadj_: 1.33, 95% CI: 1.01–1.74, p = 0.04) and *IL6R* C>T (rs4537545) (OR_adj_: 1.46, 95% CI: 1.02–2.08, p = 0.04) and both the homozygous and the heterozygous variant genotypes of *TLR2* C>T (rs1816702) (OR_adj_: 1.42, 95% CI: 1.08–1.88, p = 0.01), *TLR4* T>C (rs12377632) (OR_adj_: 1.28, 95% CI: 1.01–1.64, p = 0.05), *TLR9* −1486 T>C (rs187084) (OR_adj_: 1.29, 95% CI: 1.00–1.66, p = 0.05), *TNFRSF1A* −609 G>T (rs4149570) (OR_adj_: 1.32, 95% CI: 1.03–1.68, p = 0.03) and *IL10* C>T (rs3024505) (OR_unadj_: 1.25, 95% CI: 1.02–1.52, p = 0.03) were associated with increased risk of IBD. The homozygous variant genotype of *TLR4* T>C (rs1554973) (OR_unadj_: 0.67, 95% CI: 0.46–0.98, p = 0.04) and both the homozygous and the heterozygous variant genotypes of *IL23R* G>A (rs11209026) (OR_adj_: 0.43, 95% CI: 0.28–0.67, p = 2*10^−4^) and *PTPN22* 1858 G>A (rs2476601) (OR_adj_: 0.62, 95% CI: 0.46–0.85, p = 3*10^−3^) were associated with reduced risk of IBD (Table S1 and S2).

After Bonferroni correction for multiple testing both the homozygous and the heterozygous variant genotypes of *IL23R* G>A (rs11209026) (OR_adj_: 0.43, 95% CI: 0.28–0.67, p = 0.007) and *PTPN22* 1858 G>A (rs2476601) (OR_unadj_: 0.61, 95% CI: 0.48–0.77, p = 0.001) were associated with reduced risk of IBD.

The variant allele of the polymorphisms have been shown to increase IL-6r and IL-6 levels (*IL6R* C>T (rs4537545)), increase TLR2 levels (*TLR2* C>T (rs1816702)), increase *TNFRSF1A* expression (*TNFRSF1A* −609 G>T (rs4149570)), decrease IL-17 serum levels (*IL23R* G>A (rs11209026)) and decrease TNF-α serum levels (*PTPN22* 1858 G>A (rs2476601)), respectively (Table 2). The biological function of the polymorphisms *TLR2* A>T (rs4696480), *TLR4* T>C (rs12377632), *TLR9* −1486 T>C (rs187084), *TLR4* T>C (rs1554973) and *IL10* C>T (rs3024505) are unknown.

Thus, polymorphisms associated with higher IL-6r and IL-6 levels (*IL6R* C>T (rs4537545)), higher TLR2 levels (*TLR2* C>T (rs1816702)) and increased *TNFRSF1A* expression (*TNFRSF1A* −609 G>T (rs4149570)) were associated with increased risk of IBD. In addition, polymorphisms associated with decreased IL-17 serum levels (*IL23R* G>A (rs11209026)) and decreased TNF-α serum levels (*PTPN22* 1858 G>A (rs2476601)) were associated with reduced risk of IBD.

### Haplotype analysis

Haplotype analyses of *TLR2*, *TLR4* and *TLR9* among patients with CD, UC and IBD versus healthy controls are shown in Table S3, S4, S5, respectively. Four haplotypes in *TLR2*, three in *TLR4* and two in *TLR9* described 88%, 94% and 97% of the observed genotypes, respectively.

The *TLR2* haplotype combination 33 encompassing all the wildtype alleles (rs4696480AA, rs11938228CC, rs1816702CC and rs3804099TT) was associated with reduced risk of CD (OR: 0.19, 95% CI: 0.08–0.45, p = 0.00007) and combined CD and UC (OR: 0.32, 95% CI: 0.17–0.60, p = 0.0005).

No associations were found for *TLR4*.

The *TLR9* haplotype combination 11 (rs187084CC and rs352139GG) was associated with increased risk of UC (OR: 1.53, 95% CI: 1.03–2.28, p = 0.04). The *TLR9* haplotype combination 12 was associated with increased risk of IBD (OR: 1.35, 95% CI: 1.04–1.76, p = 0.03). Indirectly, these results support the analysis of the individual SNPs, where the variant allele of rs352139 (included in haplotype combination 22 which was used as reference) was fond to be associated with lowered risk of UC.

## Discussion

**Figure 1:**
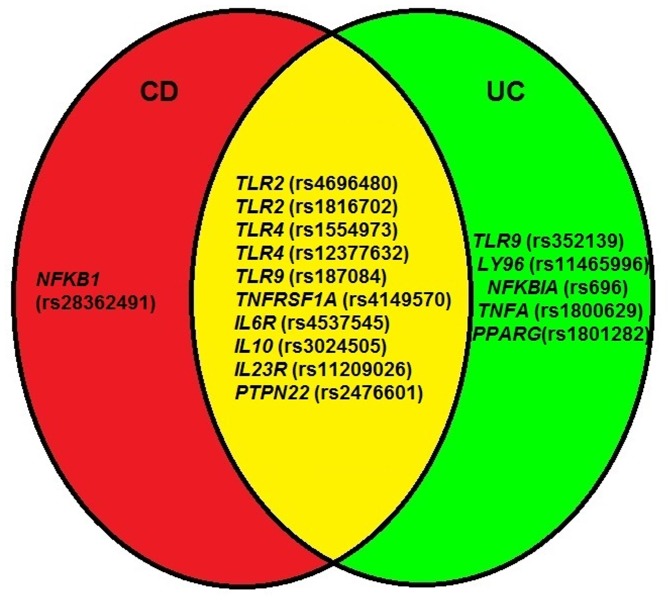
Sixteen functional single nucleotide polymorphisms (SNPs) in 13 genes involved in regulation of inflammation were found to be associated with susceptability of severe Crohn's disease (CD), ulcerative colitis (UC) or inflammatory bowel diseases (IBD). Eleven of the SNPs have not previously been reported as susceptability polymorphisms of CD, UC or IBD (*TLR2* (rs4696480 and rs1816702), *TLR4* (rs1554973 and rs12377632), *TLR9* (rs187084 and rs352139), *LY96* (rs11465996), *NFKBIA* (rs696), *TNFRSF1A* (rs4149570), *IL6R* (rs4537545) and *PTPN22* (rs2476601)).

In this study of severely ill patients, 16 functional polymorphisms in 13 genes involved in regulation of inflammation were found to be associated with CD, UC or CD and UC combined (IBD) (Figure 1). As shown in Table 2, four known susceptibility loci for CD (*PTPN22* (1858 G>A) [16]), UC (*IL10* (rs3024505 C>T) [3], [17] and *PPARG* (rs1801282 C>G) [18]) or CD and UC (*IL23R* (rs11209026 G>A) [16], [17]) were replicated. Eleven new polymorphisms associated with risk of CD (*IL6R* (rs4537545 C>T)), UC (*TLR4* (rs1554973 T>C and rs12377632 T>C), *TLR9* (1174 G>A), *LY96* (−1625 C>G), *NFKBIA* (2758 A>G) and *PTPN22* (1858 G>A)) or CD and UC (*TLR2* (−16934 A>T and rs1816702 C>T), *TLR9* (−1486 T>C) and *TNFRSF1A* (−609 G>T)) were identified. Other cohort studies of patients with CD and UC have found that other polymorphisms in the *TLR*s (toll like receptors) [19] and *TNFRSF*s (TNF receptors) [16], [17], [20], [21] were associated with CD or UC.

The biological interpretation indicates that a genetically determined higher activity of the inflammatory genes *TLR2* (rs1816702 C>T), *TNFRSF1A* (−609 G>T) and *IL6R* (rs4537545 C>T) was associated with increased risk of CD and lower activity of the inflammatory genes *NFKB1* (NFκB) (−94ins/del ATTG), *IL23R* (rs11209026 G>A) and *PTPN22* (1858 G>A) was associated with reduced risk of CD (Table 2). The picture was less clear for UC. A genetically determined higher activity of the inflammatory genes *TNFRSF1A* (−609 G>T), *PPARG* (rs1801282 C>G) and *TLR2* (rs1816702 C>T) was associated with increased risk of UC and lower activity of the inflammatory genes *IL23R* (rs11209026 G>A) and *PTPN22* (1858 G>A) was associated with reduced risk of UC. In contrast, a genetically determined higher activity of the NFκB inhibitor IκBα (*NFKBIA* 2758 A>G) and lower activity of *TLR9* (haplotype 11 (−1486CC and 1174GG)) were associated with increased risk of UC. Furthermore, a genetically determined higher activity of *LY96* (−1625 C>G) and *TNFA* (−308 G>A) was associated with reduced risk of UC. However, the risk of CD and UC seem to have shared mechanisms through the *TLR2* (rs1816702 C>T), *TNFRSF1A* (−609 G>T), *IL23R* (rs11209026 G>A) and *PTPN22* (1858 G>A) polymorphisms confirming that the inflammatory pathways are involved in risk of both CD and UC [16], [17].

This study was unable to confirm the associations between the variant allele of *IL10* (rs3024505 C>T) and increased risk of CD [3], [16] or the associations between the variant allele of *CD14* (−159 G>A) and increased risk of CD, UC and IBD [22]. This study may be underpowered to detect an association in *IL10* (rs3024505 C>T), as a GWA study with more than six thousands CD cases and fifteen thousands control found that the varaint allele was associated with increased risk of CD but with an odds ratio of 1.12 [16]. However, *IL10* has previously been found to be associated with risk of CD and UC in the Danish population [3]. The difference of association in *CD14* (−159 G>A) may be due to genetic differences between the Korean and Danish population.

The variant allele in *NFKBIA* (IκBα) (2758 A>G) [23]–[25] and *TNFA* (−308 G>A) were associated with higher and lower risk of UC, respectively, and the deletion polymorphism in *NFKB1* (NFκB) (−94ins/del ATTG) was associated with lower risk of CD in our cohort study. However, there seems to be no consensus regarding these polymorphisms in other cohort studies [24]–[27]. TLR5stop (1174 C>T) has been found to be associated with reduced risk of CD in a Jewish cohort [28] but not in a non-Jewish and German cohort [28], [29]. No association with TLR5stop was found in this cohort study.

No associations were found for *IL1B* (−31 T>C) [3], *IL-10* (−592 C>A) [3] or *PPARG* (rs1801282 C>G) [18] in accordance with our previous Danish IBD cohort study using independent cases but the same control group [3]. The other polymorphisms studied in *MAP3K14* (rs7222094 T>C), *SUMO4* (163 T>C), *TNFAIP3* (rs6927172 C>G), *IL1RN* (rs4251961 T>C), *IL4R* (rs1805010 A>G), *IL6* (−6331 T>C), *IL17A* (197G>A), *IFNG* (874 T>A), *TGFB1* (−509 C>T) and *NLRP3* (rs4612666 C>T) were not statistically associated with CD, UC or IBD in our cohort. None of the polymorphisms studied in these genes have been reported to be associated with CD or UC. We can not exclude that we did not find associations between these polymorphisms and risk of disease due to lack of power in this cohort.

The results in this study should be interpreted with care. *TLR2* (rs4696480 A>T), *TLR4* (rs1554973 T>C), *TLR9* (1174 G>A) and *TGFB1* (−509 C>T) were not in Hardy-Weinberg equilibrium among the healthy controls which is probable due to chance because of the number of polymorphisms analyzed. When corrected adequately for multiple testing they did not deviate from Hardy-Weinberg equilibrium. In the light of the obtained P-values and the number of statistical tests performed, we cannot exclude that some of our positive findings may be due to chance. If the results were corrected for multiple testing only the well known susceptibility polymorphisms in *IL23R* (rs11209026 G>A) and *PTPN22* (1858 G>A) were associated with reduced risk of both CD and IBD. We successfully tested 37 polymorphisms and, assuming a 5% acceptance level, two would be expected to be associated with susceptibility by pure chance. In this study 16 polymorphisms were found to be associated with susceptibility and the found associations were biologically plausible. A major strength was that this clinically homogeneous and well-characterised cohort was rather large including 1035 patients with IBD and 795 healthy controls. All the patients were considered for anti-TNF treatment and were therefore considered to have a severe disease course. Genetic determinants may be expected to be strong among severely ill cases [30].

In conclusion, 16 functional SNPs in 13 genes involved in regulation of inflammation were found to be associated with susceptibility of severe CD, UC or IBD. Eleven of the SNPs have not previously been reported as susceptibility polymorphisms of CD, UC or IBD (Figure 1) although other polymorphisms in most of these genes have previously been associated with susceptibility of CD, UC or IBD. Our results suggest that genetically determined high inflammatory response was associated with increased risk of CD and the large number of polymorphisms in the *TLRs* associated with risk of CD or UC support that the host microbial composition, diet or environmental molecules in the gut are important factors driving the inflammatory response in genetically susceptible individuals.

## Supporting Information

Table S1 **Odds ratios (OR) (unadjusted) for genotypes studied among healthy controls and patients with Crohn's disease (CD), ulcerative colitis (UC) and combined inflammatory bowel disease (IBD).**
(DOC)

Table S2 **Odds ratios (OR) (adjusted for age, sex and smoking status) for genotypes studied among healthy controls and patients with Crohn's disease (CD), ulcerative colitis (UC) and combined inflammatory bowel disease (IBD).**
(DOC)

Table S3 **Association of the** ***TLR2*** **haplotype combinations and risk of Crohn's disease (CD), ulcerative colitis (UC) and all inflammatory bowel disease (IBD).**
(DOC)

Table S4 **Association between** ***TLR4*** **haplotype combinations and risk of Crohn's disease (CD), ulcerative colitis (UC) and all inflammatory bowel disease (IBD).**
(DOC)

Table S5 **Association between** ***TLR9*** **haplotype combinations and risk of Crohn's disease (CD), ulcerative colitis (UC) and all inflammatory bowel disease (IBD).**
(DOC)
