# Supplementary material for: Polymorphisms in the Inflammatory Pathway Genes TLR2, TLR4, TLR9, LY96, NFKBIA, NFKB1, TNFA, TNFRSF1A, IL6R, IL10, IL23R, PTPN22, and PPARG Are Associated with Susceptibility of Inflammatory Bowel Disease in a Danish Cohort
Source: PLoS One. 2014 Jun 27;9(6):e98815. doi: 10.1371/journal.pone.0098815 (PMC4074037; doi:10.1371/journal.pone.0098815)
Supplement: Table S2 — Odds ratios (OR) (adjusted for age, sex and smoking status) for genotypes studied among healthy controls and patients with Crohn's disease (CD), ulcerative colitis (UC) and combined inflammatory bowel disease (IBD). (DOC) [file pone.0098815.s002.doc]

| **Table S2:** Odds ratios (OR) (adjusted for age, sex and smoking status) for genotypes studied among healthy controls and patients with Crohn´s disease (CD), ulcerative colitis (UC) and combined inflammatory bowel disease (IBD). | | | | | | | | | | |
| --- | --- | --- | --- | --- | --- | --- | --- | --- | --- | --- |
|  | |  |  |  | **Crohns disease (CD)*** | | **Ulcerative colitis (UC)*** | | **Inflammatory bowel disease (IBD)*** | |
| Gene (rs-number) | | **N_CD_** | **N_UC_** | **N_Control_** | Crude  OR (95% CI) | p-value | Crude  OR (95% CI) | p-value | Crude  OR (95% CI) | p-value |
| *TLR2* (rs3804099) | | |  |  |  |  |  |  |  |  |
|  | TT | 190 | 115 | 241 |  |  |  |  |  |  |
|  | TC | 281 | 215 | 393 | 0.87 (0.62-1.21) | 0.40 | 0.96 (0.67-1.38) | 0.81 | 0.92 (0.71-1.22) | 0.59 |
|  | CC | 140 | 75 | 144 | 1.23 (0.82-1.84) | 0.32 | 0.79 (0.48-1.30) | 0.36 | 1.12 (0.79-1.58) | 0.52 |
|  | TC or CC | 421 | 290 | 537 | 0.96 (0.71-1.31) | 0.81 | 0.91 (0.65-1.29) | 0.61 | 0.98 (0.76-1.26) | 0.86 |
| *TLR2* (rs11938228) | | |  |  |  |  |  |  |  |  |
|  | CC | 272 | 168 | 327 |  |  |  |  |  |  |
|  | CA | 258 | 190 | 368 | 0.79 (0.58-1.07) | 0.12 | 0.89 (0.62-1.26) | 0.49 | 0.82 (0.63-1.05) | 0.12 |
|  | AA | 82 | 47 | 76 | 1.24 (0.77-1.99) | 0.38 | 1.14 (0.66-1.97) | 0.64 | 1.11 (0.74-1.66) | 0.61 |
|  | CA or AA | 340 | 237 | 444 | 0.86 (0.64-1.15) | 0.30 | 0.93 (0.66-1.29) | 0.66 | 0.87 (0.68-1.10) | 0.24 |
| *TLR2* (rs1816702) | | |  |  |  |  |  |  |  |  |
|  | CC | 457 | 289 | 599 |  |  |  |  |  |  |
|  | CT | 130 | 108 | 148 | 1.16 (0.82-1.65) | 0.40 | 1.65 (1.13-2.40) | 0.01 | 1.36 (1.02-1.81) | 0.04 |
|  | TT | 18 | 3 | 10 | 2.80 (1.03-7.62) | 0.04 | 1.45 (0.38-5.55) | 0.59 | 2.41 (0.98-5.92) | 0.06 |
|  | CT or TT | 148 | 111 | 158 | 1.26 (0.90-1.76) | 0.18 | 1.63 (1.13-2.36) | 0.009 | 1.42 (1.08-1.88) | 0.01 |
| *TLR2* (rs4696480) | | |  |  |  |  |  |  |  |  |
|  | AA | 147 | 99 | 199 |  |  |  |  |  |  |
|  | AT | 323 | 202 | 417 | 1.04 (0.73-1.48) | 0.81 | 0.99 (0.66-1.49) | 0.96 | 1.04 (0.78-1.39) | 0.81 |
|  | TT | 150 | 104 | 155 | 1.26 (0.82-1.92) | 0.29 | 1.38 (0.86-2.21) | 0.19 | 1.27 (0.89-1.80) | 0.18 |
|  | AT or TT | 473 | 306 | 572 | 1.10 (0.79-1.54) | 0.58 | 1.09 (0.74-1.61) | 0.66 | 1.10 (0.83-1.45) | 0.51 |
| *TLR4* (rs5030728) | | |  |  |  |  |  |  |  |  |
|  | GG | 304 | 204 | 359 |  |  |  |  |  |  |
|  | GA | 252 | 151 | 323 | 0.98 (0.73-1.33) | 0.92 | 1.02 (0.72-1.44) | 0.92 | 1.00 (0.78-1.28) | 0.99 |
|  | AA | 64 | 53 | 78 | 1.08 (0.67-1.75) | 0.75 | 1.19 (0.70-2.01) | 0.53 | 1.10 (0.75-1.64) | 0.62 |
|  | GA or AA | 316 | 204 | 401 | 1.00 (0.75-1.33) | 0.98 | 1.05 (0.76-1.46) | 0.76 | 1.02 (0.80-1.29) | 0.87 |
| *TLR4* (rs1554973) | | |  |  |  |  |  |  |  |  |
|  | TT | 368 | 245 | 440 |  |  |  |  |  |  |
|  | TC | 207 | 138 | 272 | 0.87 (0.64-1.19) | 0.38 | 0.73 (0.52-1.05) | 0.09 | 0.85 (0.66-1.10) | 0.21 |
|  | CC | 37 | 21 | 62 | 0.84 (0.47-1.48) | 0.54 | 0.40 (0.18-0.86) | 0.02 | 0.66 (0.41-1.07) | 0.09 |
|  | TC or CC | 244 | 159 | 334 | 0.87 (0.65-1.16) | 0.33 | 0.67 (0.48-0.94) | 0.02 | 0.82 (0.64-1.04) | 0.10 |
| *TLR4* (rs12377632) | | |  |  |  |  |  |  |  |  |
|  | TT | 229 | 149 | 306 |  |  |  |  |  |  |
|  | TC | 280 | 193 | 358 | 1.29 (0.95-1.77) | 0.11 | 1.47 (1.02-2.10) | 0.04 | 1.33 (1.03-1.72) | 0.03 |
|  | CC | 96 | 63 | 102 | 1.10 (0.71-1.71) | 0.68 | 1.28 (0.76-2.14) | 0.35 | 1.14 (0.79-1.65) | 0.49 |
|  | TC or CC | 376 | 256 | 460 | 1.24 (0.93-1.67) | 0.15 | 1.42 (1.01-2.00) | 0.04 | 1.28 (1.01-1.64) | 0.05 |
| *TLR5* (rs5744168) | | |  |  |  |  |  |  |  |  |
|  | CC | 538 | 357 | 672 |  |  |  |  |  |  |
|  | CT | 77 | 49 | 94 | 1.03 (0.67-1.57) | 0.90 | 0.95 (0.58-1.57) | 0.85 | 1.00 (0.70-1.43) | 0.99 |
|  | TT | 6 | 2 | 5 | 1.62 (0.30-8.79) | 0.58 | 0.82 (0.09-7.87) | 0.86 | 1.42 (0.34-5.86) | 0.63 |
|  | CT or TT | 83 | 51 | 99 | 1.05 (0.69-1.60) | 0.81 | 0.95 (0.58-1.54) | 0.83 | 1.02 (0.72-1.45) | 0.90 |
| *TLR9* (rs187084) | | |  |  |  |  |  |  |  |  |
|  | TT | 189 | 126 | 262 |  |  |  |  |  |  |
|  | TC | 313 | 200 | 366 | 1.29 (0.93-1.79) | 0.13 | 1.41 (0.96-2.06) | 0.08 | 1.30 (0.99-1.71) | 0.06 |
|  | CC | 113 | 81 | 142 | 1.20 (0.79-1.81) | 0.40 | 1.39 (0.87-2.22) | 0.16 | 1.24 (0.88-1.75) | 0.21 |
|  | TC or CC | 426 | 281 | 508 | 1.26 (0.93-1.72) | 0.14 | 1.40 (0.98-2.00) | 0.06 | 1.29 (1.00-1.66) | 0.05 |
| *TLR9* (rs352139) | | |  |  |  |  |  |  |  |  |
|  | GG | 193 | 146 | 255 |  |  |  |  |  |  |
|  | GA | 307 | 196 | 347 | 1.12 (0.81-1.55) | 0.48 | 1.10 (0.76-1.57) | 0.62 | 1.11 (0.85-1.44) | 0.46 |
|  | AA | 119 | 64 | 167 | 0.78 (0.52-1.18) | 0.24 | 0.62 (0.38-1.02) | 0.06 | 0.75 (0.53-1.05) | 0.09 |
|  | GA or AA | 426 | 260 | 514 | 1.01 (0.74-1.36) | 0.96 | 0.94 (0.67-1.32) | 0.72 | 0.99 (0.78-1.27) | 0.93 |
| *LY96* (rs11465996) | | |  |  |  |  |  |  |  |  |
|  | CC | 291 | 208 | 344 |  |  |  |  |  |  |
|  | CG | 269 | 151 | 337 | 0.85 (0.63-1.15) | 0.30 | 0.83 (0.59-1.18) | 0.31 | 0.84 (0.65-1.07) | 0.16 |
|  | GG | 62 | 50 | 81 | 0.89 (0.55-1.44) | 0.64 | 1.11 (0.66-1.88) | 0.68 | 0.99 (0.67-1.46) | 0.95 |
|  | CG or GG | 331 | 201 | 418 | 0.86 (0.65-1.15) | 0.30 | 0.89 (0.64-1.23) | 0.48 | 0.87 (0.68-1.10) | 0.23 |
| *CD14* (rs2569190) | | |  |  |  |  |  |  |  |  |
|  | GG | 166 | 117 | 236 |  |  |  |  |  |  |
|  | GA | 314 | 201 | 360 | 1.21 (0.86-1.69) | 0.28 | 1.21 (0.82-1.77) | 0.34 | 1.20 (0.91-1.58) | 0.21 |
|  | AA | 138 | 90 | 170 | 1.17 (0.78-1.76) | 0.44 | 1.09 (0.69-1.73) | 0.71 | 1.12 (0.80-1.57) | 0.50 |
|  | GA or AA | 452 | 291 | 530 | 1.20 (0.87-1.64) | 0.27 | 1.17 (0.81-1.68) | 0.40 | 1.17 (0.90-1.52) | 0.23 |
| *MAP3K14* (rs7222094) | | |  |  |  |  |  |  |  |  |
|  | TT | 191 | 131 | 235 |  |  |  |  |  |  |
|  | TC | 303 | 202 | 383 | 1.02 (0.73-1.42) | 0.92 | 1.01 (0.70-1.47) | 0.95 | 1.00 (0.76-1.32) | 0.97 |
|  | CC | 118 | 70 | 147 | 0.99 (0.66-1.50) | 0.97 | 0.82 (0.50-1.32) | 0.41 | 0.92 (0.65-1.30) | 0.63 |
|  | TC or CC | 421 | 272 | 530 | 1.01 (0.74-1.38) | 0.95 | 0.95 (0.67-1.35) | 0.79 | 0.98 (0.76-1.27) | 0.88 |
| *SUMO4* (rs237025) | | |  |  |  |  |  |  |  |  |
|  | TT | 185 | 107 | 215 |  |  |  |  |  |  |
|  | TC | 295 | 210 | 362 | 1.02 (0.73-1.43) | 0.89 | 0.90 (0.61-1.32) | 0.58 | 0.95 (0.72-1.25) | 0.70 |
|  | CC | 141 | 92 | 195 | 0.98 (0.66-1.46) | 0.92 | 0.94 (0.61-1.46) | 0.78 | 0.92 (0.66-1.27) | 0.60 |
|  | TC or CC | 436 | 302 | 557 | 1.01 (0.74-1.38) | 0.96 | 0.91 (0.64-1.30) | 0.61 | 0.94 (0.72-1.22) | 0.62 |
| *NFKBIA* (rs696) | | |  |  |  |  |  |  |  |  |
|  | GG | 247 | 136 | 298 |  |  |  |  |  |  |
|  | GA | 297 | 215 | 366 | 0.91 (0.67-1.23) | 0.53 | 1.43 (1.00-2.06) | 0.05 | 1.06 (0.82-1.37) | 0.64 |
|  | AA | 78 | 58 | 101 | 0.84 (0.53-1.34) | 0.47 | 1.52 (0.92-2.51) | 0.11 | 1.07 (0.73-1.56) | 0.73 |
|  | GA or AA | 375 | 273 | 467 | 0.89 (0.67-1.19) | 0.44 | 1.45 (1.03-2.05) | 0.03 | 1.06 (0.83-1.36) | 0.62 |
| *NFKBIA* (rs17103265) | | |  |  |  |  |  |  |  |  |
|  | TT | 613 | 404 | 776 |  |  |  |  |  |  |
|  | T/del | 1 | 3 | 1 | 2.07(0.11-40.83) | 0.63 | 4.18(0.26-67.93) | 0.31 | 3.08(0.26-35.96) | 0.37 |
|  | del/del | 1 | 0 | 0 | - | - | - | - | - | - |
|  | T/del or del/del | 2 | 3 | 1 | 3.51(0.27-45.19) | 0.34 | 4.18(0.26-67.93) | 0.31 | 4.14(0.41-41.76) | 0.23 |
| *NFKB1* (rs28362491) | | |  |  |  |  |  |  |  |  |
|  | Ins/ins | 250 | 146 | 269 |  |  |  |  |  |  |
|  | Ins/del | 289 | 193 | 376 | 0.88 (0.65-1.20) | 0.43 | 0.91 (0.63-1.30) | 0.59 | 0.90 (0.69-1.16) | 0.42 |
|  | Deldel | 83 | 69 | 122 | 0.67 (0.43-1.05) | 0.08 | 1.12 (0.71-1.78) | 0.62 | 0.88 (0.61-1.25) | 0.47 |
|  | Ins/del or del/del | 372 | 262 | 498 | 0.83 (0.62-1.11) | 0.21 | 0.96 (0.69-1.35) | 0.83 | 0.89 (0.70-1.14) | 0.37 |
| *TNFA* (rs1800629) | | |  |  |  |  |  |  |  |  |
|  | GG | 426 | 302 | 527 |  |  |  |  |  |  |
|  | GA | 174 | 94 | 223 | 0.84 (0.61-1.16) | 0.30 | 0.79 (0.54-1.14) | 0.21 | 0.82 (0.63-1.07) | 0.15 |
|  | AA | 22 | 13 | 25 | 1.01 (0.48-2.12) | 0.98 | 0.99 (0.40-2.41) | 0.98 | 0.90 (0.48-1.71) | 0.75 |
|  | GA or AA | 196 | 107 | 248 | 0.86 (0.64-1.17) | 0.34 | 0.81 (0.57-1.15) | 0.24 | 0.83 (0.65-1.07) | 0.16 |
| *TNFA* (rs361525) | | |  |  |  |  |  |  |  |  |
|  | GG | 556 | 380 | 708 |  |  |  |  |  |  |
|  | GA | 60 | 21 | 60 | 1.09 (0.66-1.80) | 0.74 | 0.69 (0.35-1.37) | 0.29 | 0.93 (0.60-1.43) | 0.74 |
|  | AA | 2 | 2 | 3 | 3.37(0.44-25.69) | 0.24 | 1.73(0.25-12.08) | 0.58 | 2.49(0.45-13.80) | 0.30 |
|  | GA or AA | 62 | 23 | 63 | 1.15 (0.70-1.87) | 0.58 | 0.75 (0.40-1.43) | 0.39 | 0.98 (0.64-1.50) | 0.93 |
| *TNFRSF1A* (rs4149570) | | |  |  |  |  |  |  |  |  |
|  | GG | 212 | 140 | 307 |  |  |  |  |  |  |
|  | GT | 291 | 186 | 355 | 1.21 (0.88-1.65) | 0.24 | 1.28 (0.89-1.84) | 0.19 | 1.20 (0.93-1.56) | 0.16 |
|  | TT | 106 | 74 | 109 | 1.84 (1.19-2.84) | 0.006 | 1.85 (1.16-2.97) | 0.01 | 1.72 (1.21-2.46) | 0.003 |
|  | GT or TT | 397 | 260 | 464 | 1.33 (0.99-1.80) | 0.06 | 1.41 (1.00-1.98) | 0.05 | 1.32 (1.03-1.68) | 0.03 |
| *TNFAIP3* (rs6927172) | | |  |  |  |  |  |  |  |  |
|  | CC | 381 | 237 | 473 |  |  |  |  |  |  |
|  | CG | 206 | 143 | 264 | 1.27 (0.93-1.72) | 0.13 | 1.08 (0.76-1.53) | 0.65 | 1.19 (0.93-1.54) | 0.17 |
|  | GG | 27 | 24 | 40 | 1.02 (0.50-2.09) | 0.95 | 1.40 (0.69-2.85) | 0.34 | 1.18 (0.68-2.07) | 0.56 |
|  | CG or GG | 233 | 167 | 304 | 1.24 (0.92-1.66) | 0.16 | 1.12 (0.81-1.56) | 0.49 | 1.19 (0.93-1.52) | 0.16 |
| *IL1B* (rs4848306) | | |  |  |  |  |  |  |  |  |
|  | GG | 179 | 129 | 246 |  |  |  |  |  |  |
|  | GA | 330 | 205 | 373 | 1.36 (0.98-1.89) | 0.07 | 0.95 (0.66-1.35) | 0.77 | 1.12 (0.85-1.46) | 0.42 |
|  | AA | 114 | 75 | 151 | 1.32 (0.87-2.00) | 0.19 | 0.64 (0.39-1.05) | 0.08 | 0.95 (0.67-1.34) | 0.76 |
|  | GA or AA | 444 | 280 | 524 | 1.35 (0.99-1.84) | 0.06 | 0.86 (0.61-1.21) | 0.38 | 1.07 (0.83-1.38) | 0.61 |
| *IL1B* (rs1143623) | | |  |  |  |  |  |  |  |  |
|  | GG | 334 | 218 | 401 |  |  |  |  |  |  |
|  | GC | 246 | 160 | 316 | 0.82 (0.60-1.10) | 0.18 | 0.99 (0.70-1.40) | 0.96 | 0.88 (0.69-1.13) | 0.32 |
|  | CC | 39 | 30 | 55 | 0.77 (0.43-1.38) | 0.37 | 1.72 (0.96-3.08) | 0.07 | 1.17 (0.73-1.86) | 0.51 |
|  | GC or CC | 285 | 190 | 371 | 0.81 (0.61-1.08) | 0.15 | 1.09 (0.79-1.50) | 0.61 | 0.92 (0.73-1.17) | 0.49 |
| *IL1B* (rs1143627) | | |  |  |  |  |  |  |  |  |
|  | TT | 281 | 177 | 340 |  |  |  |  |  |  |
|  | TC | 279 | 187 | 339 | 0.92 (0.68-1.24) | 0.57 | 0.98 (0.69-1.39) | 0.91 | 0.94 (0.73-1.21) | 0.64 |
|  | CC | 60 | 47 | 97 | 0.68 (0.42-1.10) | 0.11 | 1.18 (0.71-1.97) | 0.52 | 0.91 (0.62-1.34) | 0.63 |
|  | TC or CC | 339 | 234 | 436 | 0.86 (0.65-1.15) | 0.31 | 1.02 (0.74-1.41) | 0.90 | 0.94 (0.74-1.19) | 0.58 |
| *IL1RN* (rs4251961) | | |  |  |  |  |  |  |  |  |
|  | TT | 218 | 138 | 298 |  |  |  |  |  |  |
|  | TC | 315 | 201 | 360 | 1.14 (0.84-1.56) | 0.39 | 1.14 (0.80-1.63) | 0.47 | 1.17 (0.90-1.51) | 0.24 |
|  | CC | 86 | 72 | 112 | 1.01 (0.64-1.59) | 0.97 | 1.24 (0.76-2.03) | 0.38 | 1.10 (0.76-1.59) | 0.61 |
|  | TC or CC | 401 | 273 | 472 | 1.11 (0.83-1.50) | 0.47 | 1.16 (0.83-1.63) | 0.38 | 1.15 (0.90-1.47) | 0.25 |
| *IL4R* (rs1805010) | | |  |  |  |  |  |  |  |  |
|  | AA | 188 | 113 | 209 |  |  |  |  |  |  |
|  | AG | 300 | 207 | 410 | 0.80 (0.57-1.12) | 0.19 | 0.95 (0.64-1.41) | 0.81 | 0.87 (0.66-1.15) | 0.32 |
|  | GG | 130 | 87 | 157 | 0.78 (0.52-1.19) | 0.25 | 1.14 (0.71-1.83) | 0.58 | 0.92 (0.65-1.30) | 0.65 |
|  | AG or GG | 430 | 294 | 567 | 0.80 (0.58-1.09) | 0.16 | 1.00 (0.69-1.46) | 0.98 | 0.88 (0.68-1.15) | 0.36 |
| *IL6* (rs10499563) | | |  |  |  |  |  |  |  |  |
|  | TT | 411 | 255 | 476 |  |  |  |  |  |  |
|  | TC | 186 | 134 | 259 | 0.84 (0.62-1.15) | 0.27 | 0.85 (0.60-1.20) | 0.35 | 0.85 (0.66-1.09) | 0.21 |
|  | CC | 23 | 19 | 35 | 1.03 (0.52-2.06) | 0.93 | 0.74 (0.32-1.75) | 0.50 | 0.92 (0.51-1.65) | 0.79 |
|  | TC or CC | 209 | 153 | 294 | 0.86 (0.64-1.16) | 0.33 | 0.83 (0.60-1.17) | 0.29 | 0.86 (0.67-1.09) | 0.22 |
| *IL6R* (rs4537545) | | |  |  |  |  |  |  |  |  |
|  | CC | 211 | 143 | 289 |  |  |  |  |  |  |
|  | CT | 310 | 201 | 369 | 1.18 (0.86-1.62) | 0.31 | 1.05 (0.73-1.50) | 0.80 | 1.09 (0.84-1.41) | 0.53 |
|  | TT | 97 | 65 | 117 | 1.73 (1.12-2.66) | 0.01 | 1.33 (0.83-2.14) | 0.24 | 1.45 (1.02-2.08) | 0.04 |
|  | CT or TT | 407 | 266 | 486 | 1.29 (0.96-1.74) | 0.09 | 1.11 (0.79-1.56) | 0.53 | 1.17 (0.91-1.50) | 0.21 |
| *IL10* (rs1800872) | | |  |  |  |  |  |  |  |  |
|  | CC | 370 | 257 | 482 |  |  |  |  |  |  |
|  | CA | 226 | 131 | 258 | 1.15 (0.85-1.55) | 0.37 | 0.94 (0.66-1.33) | 0.71 | 1.06 (0.82-1.36) | 0.65 |
|  | AA | 21 | 20 | 35 | 0.76 (0.36-1.61) | 0.47 | 1.11 (0.52-2.35) | 0.79 | 0.90 (0.50-1.63) | 0.73 |
|  | CA or AA | 247 | 151 | 293 | 1.10 (0.82-1.47) | 0.52 | 0.96 (0.69-1.33) | 0.79 | 1.04 (0.82-1.32) | 0.75 |
| *IL10* (rs3024505) | | |  |  |  |  |  |  |  |  |
|  | CC | 404 | 244 | 518 |  |  |  |  |  |  |
|  | CT | 189 | 135 | 221 | 0.98 (0.71-1.34) | 0.88 | 1.25 (0.88-1.77) | 0.22 | 1.07 (0.82-1.38) | 0.62 |
|  | TT | 28 | 27 | 22 | 1.51 (0.69-3.31) | 0.30 | 1.95 (0.88-4.32) | 0.10 | 1.64 (0.87-3.11) | 0.13 |
|  | CT or TT | 217 | 162 | 243 | 1.02 (0.75-1.38) | 0.89 | 1.31 (0.94-1.84) | 0.11 | 1.12 (0.87-1.43) | 0.38 |
| *IL17A* (rs2275913) | | |  |  |  |  |  |  |  |  |
|  | GG | 275 | 170 | 340 |  |  |  |  |  |  |
|  | GA | 269 | 184 | 336 | 1.14 (0.84-1.54) | 0.40 | 1.20 (0.85-1.70) | 0.29 | 1.15 (0.89-1.47) | 0.29 |
|  | AA | 73 | 53 | 95 | 0.93 (0.57-1.50) | 0.75 | 1.01 (0.60-1.71) | 0.97 | 0.98 (0.67-1.46) | 0.94 |
|  | GA or AA | 342 | 237 | 431 | 1.09 (0.82-1.46) | 0.55 | 1.16 (0.83-1.61) | 0.38 | 1.11 (0.88-1.41) | 0.39 |
| *IL23R* (rs11209026) | | |  |  |  |  |  |  |  |  |
|  | GG | 591 | 380 | 680 |  |  |  |  |  |  |
|  | GA | 32 | 31 | 89 | 0.39 (0.22-0.70) | 0.001 | 0.55 (0.30-0.99) | 0.05 | 0.45 (0.29-0.70) | 0.0005 |
|  | AA | 0 | 0 | 5 | - | - | - | - | - | - |
|  | GA or AA | 32 | 31 | 94 | 0.38 (0.21-0.67) | 0.0009 | 0.52 (0.29-0.94) | 0.03 | 0.43 (0.28-0.67) | 0.0002 |
| *IFNG* (rs2430561) | | |  |  |  |  |  |  |  |  |
|  | TT | 173 | 112 | 199 |  |  |  |  |  |  |
|  | TA | 276 | 201 | 398 | 0.86 (0.61-1.21) | 0.38 | 1.04 (0.71-1.54) | 0.83 | 0.94 (0.71-1.25) | 0.66 |
|  | AA | 159 | 87 | 161 | 1.31 (0.88-1.94) | 0.19 | 1.07 (0.66-1.72) | 0.79 | 1.21 (0.87-1.70) | 0.26 |
|  | TA or AA | 435 | 288 | 559 | 0.99 (0.72-1.36) | 0.93 | 1.05 (0.73-1.52) | 0.79 | 1.02 (0.78-1.33) | 0.89 |
| *TGFB1* (rs1800469) | | |  |  |  |  |  |  |  |  |
|  | CC | 308 | 212 | 383 |  |  |  |  |  |  |
|  | CT | 255 | 161 | 297 | 1.05 (0.77-1.41) | 0.77 | 1.11 (0.79-1.57) | 0.54 | 1.10 (0.85-1.41) | 0.47 |
|  | TT | 56 | 35 | 86 | 0.68 (0.40-1.14) | 0.14 | 1.07 (0.62-1.83) | 0.81 | 0.83 (0.55-1.25) | 0.37 |
|  | CT or TT | 311 | 196 | 383 | 0.96 (0.73-1.28) | 0.80 | 1.10 (0.80-1.53) | 0.55 | 1.04 (0.82-1.31) | 0.77 |
| *PTPN22* (rs2476601) | | |  |  |  |  |  |  |  |  |
|  | GG | 533 | 338 | 588 |  |  |  |  |  |  |
|  | GA | 85 | 68 | 166 | 0.60 (0.41-0.88) | 0.009 | 0.71 (0.46-1.09) | 0.11 | 0.64 (0.47-0.87) | 0.005 |
|  | AA | 2 | 4 | 11 | 0.17 (0.02-1.39) | 0.10 | 0.72 (0.15-3.46) | 0.68 | 0.38 (0.10-1.50) | 0.17 |
|  | GA or AA | 87 | 72 | 177 | 0.57 (0.39-0.83) | 0.004 | 0.71 (0.47-1.08) | 0.11 | 0.62 (0.46-0.85) | 0.003 |
| *PPARG* (rs1801282) | | |  |  |  |  |  |  |  |  |
|  | CC | 456 | 277 | 548 |  |  |  |  |  |  |
|  | CG | 149 | 115 | 207 | 0.76 (0.54-1.06) | 0.11 | 1.02 (0.70-1.47) | 0.93 | 0.86 (0.66-1.13) | 0.29 |
|  | GG | 9 | 15 | 14 | 0.66 (0.25-1.78) | 0.41 | 1.52 (0.51-4.51) | 0.45 | 0.95 (0.41-2.20) | 0.91 |
|  | CG or GG | 158 | 130 | 221 | 0.75 (0.54-1.04) | 0.09 | 1.05 (0.73-1.50) | 0.80 | 0.87 (0.67-1.13) | 0.31 |
| *NLRP3* (rs4612666) | | |  |  |  |  |  |  |  |  |
|  | CC | 355 | 226 | 435 |  |  |  |  |  |  |
|  | CT | 221 | 159 | 280 | 0.92 (0.68-1.25) | 0.60 | 1.07 (0.76-1.51) | 0.68 | 1.01 (0.78-1.29) | 0.96 |
|  | TT | 42 | 23 | 53 | 0.80 (0.44-1.44) | 0.45 | 0.90 (0.45-1.83) | 0.78 | 0.81 (0.50-1.32) | 0.40 |
|  | CT or TT | 263 | 182 | 333 | 0.90 (0.68-1.20) | 0.49 | 1.05 (0.76-1.45) | 0.78 | 0.97 (0.77-1.24) | 0.83 |
| *Adjusted for age, sex and smoking status. | | | | | | | | | | |
