# Supplementary material for: Polymorphisms in the Inflammatory Pathway Genes TLR2, TLR4, TLR9, LY96, NFKBIA, NFKB1, TNFA, TNFRSF1A, IL6R, IL10, IL23R, PTPN22, and PPARG Are Associated with Susceptibility of Inflammatory Bowel Disease in a Danish Cohort
Source: PLoS One. 2014 Jun 27;9(6):e98815. doi: 10.1371/journal.pone.0098815 (PMC4074037; doi:10.1371/journal.pone.0098815)
Supplement: Table S3 — Association of the TLR2 haplotype combinations and risk of Crohn's disease (CD), ulcerative colitis (UC) and all inflammatory bowel disease (IBD). (DOC) [file pone.0098815.s003.doc]

| **Table S3:** Association of the *TLR2* haplotype combinations and risk of Crohn's disease (CD), ulcerative colitis (UC) and all inflammatory bowel disease (IBD). | | | | | | | | | | | | | | | | |
| --- | --- | --- | --- | --- | --- | --- | --- | --- | --- | --- | --- | --- | --- | --- | --- | --- |
| Haplotype combinations | Haplotypes | | | |  |  |  | Crohn's disease (CD) vs Controls | | | Ulcerative colitis (UC) vs Controls | | | Inflammatory bowel disease (IBD) vs Controls | | |
|  | rs4696480 A>T | rs11938228 C>A | rs1816702 C>T | rs3804099 T>C | N**_CD_** | N**_UC_** | N**_Control_** | OR^1^ | (95% CI) | P-value | OR^1^ | (95% CI) | P-value | OR^1^ | (95% CI) | P-value |
| 11 | T:T | A:A | C:C | T:T | 81 | 44 | 74 | 1.00 | - | - | 1.00 | - | - | 1.00 | - | - |
| 22 | A:A | C:C | C:C | C:C | 70 | 36 | 73 | 0.88 | 0.56-1.38 | 0.64 | 0.83 | 0.48-1.43 | 0.58 | 0.87 | 0.58-1.32 | 0.53 |
| 33 | A:A | C:C | C:C | T:T | 7 | 11 | 34 | 0.19 | 0.08-0.45 | 0.00007 | 0.54 | 0.25-1.18 | 0.14 | 0.32 | 0.17-0.60 | 0.0005 |
| 44 | T:T | C:C | T:T | C:C | 13 | 1 | 10 | 1.19 | 0.49-2.87 | 0.82 | 0.17 | 0.02-1.36 | 0.10 | 0.84 | 0.36-1.99 | 0.82 |
|  |  |  |  |  |  |  |  |  |  |  |  |  |  |  |  |  |
| 12 | T:A | C:A | C:C | C:T | 115 | 88 | 192 | 0.55 | 0.37-0.81 | 0.003 | 0.77 | 0.49-1.21 | 0.29 | 0.64 | 0.45-0.90 | 0.01 |
| 13 | T:A | C:A | C:C | T:T | 79 | 36 | 103 | 0.70 | 0.46-1.08 | 0.13 | 0.59 | 0.35-1.00 | 0.06 | 0.67 | 0.45-0.99 | 0.05 |
| 14 | T:T | C:A | T:C | C:T | 33 | 40 | 48 | 0.63 | 0.36-1.08 | 0.10 | 1.40 | 0.80-2.46 | 0.25 | 0.92 | 0.58-1.46 | 0.72 |
|  |  |  |  |  |  |  |  |  |  |  |  |  |  |  |  |  |
| 23 | A:A | C:C | C:C | C:T | 58 | 46 | 88 | 0.60 | 0.38-0.95 | 0.04 | 0.88 | 0.52-1.47 | 0.69 | 0.71 | 0.47-1.07 | 0.10 |
| 24 | T:A | C:C | T:C | C:C | 48 | 33 | 54 | 0.81 | 0.49-1.34 | 0.45 | 1.02 | 0.58-1.82 | 1.00 | 0.90 | 0.58-1.41 | 0.73 |
|  |  |  |  |  |  |  |  |  |  |  |  |  |  |  |  |  |
| 34 | T:A | C:C | T:C | C:T | 38 | 23 | 34 | 1.02 | 0.58-1.79 | 1.00 | 1.14 | 0.60-2.17 | 0.74 | 1.08 | 0.65-1.80 | 0.80 |
| OR: Odds ratio.  ^1^OR was calculated for each haplotype combination by using the haplotype 11 as refence group. | | | | | | | | | | | | | | | | |
