# Supplementary material for: Polymorphisms in the Inflammatory Pathway Genes TLR2, TLR4, TLR9, LY96, NFKBIA, NFKB1, TNFA, TNFRSF1A, IL6R, IL10, IL23R, PTPN22, and PPARG Are Associated with Susceptibility of Inflammatory Bowel Disease in a Danish Cohort
Source: PLoS One. 2014 Jun 27;9(6):e98815. doi: 10.1371/journal.pone.0098815 (PMC4074037; doi:10.1371/journal.pone.0098815)
Supplement: Table S4 — Association between TLR4 haplotype combinations and risk of Crohn's disease (CD), ulcerative colitis (UC) and all inflammatory bowel disease (IBD). (DOC) [file pone.0098815.s004.doc]

| **Table S4:** Association between *TLR4* haplotype combinations and risk of Crohn's disease (CD), ulcerative colitis (UC) and all inflammatory bowel disease (IBD). | | | | | | | | | | | | | | | |
| --- | --- | --- | --- | --- | --- | --- | --- | --- | --- | --- | --- | --- | --- | --- | --- |
| Haplotype combinations | Haplotypes | | |  |  |  | Crohn's disease (CD) vs Controls | | | Ulcerative colitis (UC) vs Controls | | | Inflammatory bowel disease (IBD) vs Controls | | |
|  | rs12377632  T>C | rs1554973  T>C | rs5030728  G>A | N**_CD_** | N**_UC_** | N**_Control_** | OR^1^ | (95% CI) | P-value | OR^1^ | (95% CI) | P-value | OR^1^ | (95% CI) | P-value |
| 11 | C:C | T:T | G:G | 89 | 61 | 101 | 1.00 | - | - | 1.00 | - | - | 1.00 | - | - |
| 22 | T:T | T:T | A:A | 63 | 53 | 74 | 0.97 | 0.62-1.50 | 0.91 | 1.19 | 0.74-1.91 | 0.54 | 1.06 | 0.72-1.55 | 0.84 |
| 33 | T:T | C:C | G:G | 35 | 21 | 57 | 0.70 | 0.42-1.16 | 0.20 | 0.61 | 0.34-1.10 | 0.11 | 0.66 | 0.42-1.03 | 0.09 |
| 44 | T:T | T:T | G:G | 3 | 1 | 5 | 0.68 | 0.16-2.93 | 0.73 | 0.33 | 0.04-2.90 | 0.42 | 0.54 | 0.14-2.05 | 0.49 |
|  |  |  |  |  |  |  |  |  |  |  |  |  |  |  |  |
| 12 | T:C | T:T | G:A | 139 | 95 | 188 | 0.84 | 0.59-1.20 | 0.36 | 0.84 | 0.56-1.25 | 0.41 | 0.84 | 0.61-1.15 | 0.30 |
| 13 | T:C | T:C | G:G | 101 | 70 | 129 | 0.89 | 0.60-1.31 | 0.56 | 0.90 | 0.58-1.38 | 0.66 | 0.89 | 0.64-1.25 | 0.54 |
| 14 | T:C | T:T | G:G | 33 | 24 | 32 | 1.17 | 0.67-2.06 | 0.67 | 1.24 | 0.67-2.30 | 0.53 | 1.20 | 0.73-1.98 | 0.53 |
|  |  |  |  |  |  |  |  |  |  |  |  |  |  |  |  |
| 23 | T:T | T:C | G:A | 74 | 47 | 106 | 0.79 | 0.53-1.20 | 0.30 | 0.73 | 0.46-1.17 | 0.23 | 0.77 | 0.53-1.10 | 0.17 |
| 24 | T:T | T:T | G:A | 29 | 9 | 24 | 1.37 | 0.74-2.53 | 0.35 | 0.62 | 0.27-1.42 | 0.32 | 1.07 | 0.60-1.89 | 0.89 |
|  |  |  |  |  |  |  |  |  |  |  |  |  |  |  |  |
| 34 | T:T | T:C | G:G | 20 | 17 | 26 | 0.87 | 0.46-1.67 | 0.74 | 1.08 | 0.54-2.16 | 0.86 | 0.96 | 0.55-1.68 | 0.89 |
| OR: Odds ratio.  ^1^OR was calculated for each haplotype combination by using the haplotype 11 as refence group. | | | | | | | | | | | | | | | |
