# Supplementary material for: Polymorphisms in the Inflammatory Pathway Genes TLR2, TLR4, TLR9, LY96, NFKBIA, NFKB1, TNFA, TNFRSF1A, IL6R, IL10, IL23R, PTPN22, and PPARG Are Associated with Susceptibility of Inflammatory Bowel Disease in a Danish Cohort
Source: PLoS One. 2014 Jun 27;9(6):e98815. doi: 10.1371/journal.pone.0098815 (PMC4074037; doi:10.1371/journal.pone.0098815)
Supplement: Table S5 — Association between TLR9 haplotype combinations and risk of Crohn's disease (CD), ulcerative colitis (UC) and all inflammatory bowel disease (IBD). (DOC) [file pone.0098815.s005.doc]

| **Table S5:** Association between *TLR9* haplotype combinations and risk of Crohn's disease (CD), ulcerative colitis (UC) and all inflammatory bowel disease (IBD). | | | | | | | | | | | | | | |
| --- | --- | --- | --- | --- | --- | --- | --- | --- | --- | --- | --- | --- | --- | --- |
| Haplotype combinations | Haplotypes | |  |  |  | Crohn's disease (CD) vs  Controls | | | Ulcerative colitis (UC) vs  Controls | | | Inflammatory bowel disease (IBD) vs Controls | | |
|  | rs187084  -1486 T>C | rs352139  1174 G>A | N**_CD_** | N**_UC_** | N**_Control_** | OR^1^ | (95% CI) | P-value | OR^1^ | (95% CI) | P-value | OR^1^ | (95% CI) | P-value |
| 11 | C:C | G:G | 113 | 81 | 140 | 1.15 | 0.82-1.62 | 0.43 | 1.53 | 1.03-2.28 | 0.04 | 1.29 | 0.95-1.74 | 0.11 |
| 22 | T:T | A:A | 117 | 63 | 167 | 1.00 | - | - | 1.00 | - | - | 1.00 | - | - |
| 33 | T:T | G:G | 6 | 8 | 12 | 0.71 | 0.26-1.96 | 0.62 | 1.77 | 0.69-4.53 | 0.30 | 1.08 | 0.49-2.41 | 1.00 |
|  |  |  |  |  |  |  |  |  |  |  |  |  |  |  |
| 12 | C:T | G:A | 241 | 141 | 262 | 1.31 | 0.98-1.76 | 0.07 | 1.43 | 1.00-2.03 | 0.05 | 1.35 | 1.04-1.76 | 0.03 |
| 13 | C:T | G:G | 70 | 56 | 101 | 0.99 | 0.67-1.45 | 1.00 | 1.47 | 0.95-2.27 | 0.09 | 1.16 | 0.83-1.62 | 0.44 |
|  |  |  |  |  |  |  |  |  |  |  |  |  |  |  |
| 23 | T:T | G:A | 64 | 52 | 78 | 1.17 | 0.78-1.76 | 0.47 | 1.77 | 1.12-2.79 | 0.02 | 1.38 | 0.97-1.97 | 0.09 |
| The -1486C allele in combination with the 1174G allele has been shown to down regulate expression [31].  OR: Odds ratio.  ^1^OR was calculated for each haplotype combination by using the haplotype 22 as refence group. | | | | | | | | | | | | | | |
